# Descriptive Longitudinal Pilot Study: Behaviors Surrounding Feeding of Preterm Infants Who Received Extended Tube Feedings

**DOI:** 10.24966/ncp-878x/100092

**Published:** 2022-04-13

**Authors:** Rosemary White-Traut, Thao Griffith, Cheng Zheng, Joanne Lagatta, Christina Rigby-McCotter, Claire Walsh, Karen Gralton

**Affiliations:** 1 Nursing Research, Children’s Wisconsin, Milwaukee, Wisconsin, USA; 2 Women, Children and Family Health Science, College of Nursing, University of Illinois at Chicago, Chicago, Illinois, USA; 3 Department of Family and Community Health Nursing, Marcella Niehoff School of Nursing, Loyola University Chicago, Chicago, Illinois, USA; 4 Department of Biostatistics, College of Public Health, University of Nebraska Medical Center, Omaha, Nebraska, USA; 5 Divison of Neonatology, Department of Pediactrics, Medical College of Wisconsin, Milwaukee, Wisconsin, USA

## Abstract

**Purpose:**

Seventy percent of preterm infants experience challenges with oral feeding and commonly require tube feedings. Yet it is not well understood how these behaviors change over time while infants are receiving tube feedings only and through the transition to oral feedings. The purpose of this pilot study was to describe the change in behaviors surrounding feeding and with respect to advancing Post Menstrual Age (PMA) for preterm infants who received extended tube feedings during hospitalization in the Neonatal Intensive Care Unit (NICU).

**Methods and measures:**

A prospective pilot study was conducted in a NICU. We recruited thirty-five infants who were born between 28 to 32 weeks gestational age and expected to have at least two weeks of tube feedings. Infant health status and feeding progression were obtained from the medical record. Behaviors surrounding feeding included infant state, social interactive behaviors, orally directed behaviors, and hunger/satiation cues were evaluated via weekly recorded videos.

**Results:**

During the pre-feeding segment, we noted an increase over time for awake, facial gaze, gaze aversion, tongue protrusion, fussing, mouthing, vocalization, and interest in the pacifier. During the intra-feeding segment, we found an increase over time for fussing, and a decrease for eye widening, eye searching, and vocalization.

**Conclusion:**

To our knowledge, this was the first pilot study to comprehensively describe the changes in behaviors surrounding feeding over time and with respect to advancing PMA for preterm infants who received extended tube feedings during the NICU hospitalization. Infants demonstrated distinct behaviors surrounding feeding as young as 28 weeks post menstrual age. These behaviors may vary among infants and change significantly with advancing post-menstrual age. Assessment of subtle behaviors surrounding feeding is important to ensure oral feeding readiness.

## Introduction

Globally, approximately 15 million infants are born prematurely (before 37 weeks Gestational Age, GA), each year with the United States ranking within the top ten countries for preterm births [1]. Due to the lack of mature oral feeding skills (suck-breathe-swallow co-ordination) and oral feeding readiness for safe and efficient feeding, up to 70% of preterm infants experience challenges with oral feeding [2]. Therefore, while these infants mature and learn to oral feed safely and efficiently, tube feedings (either nasogastric or orogastric) are temporarily necessary to provide adequate caloric intake and support energy conservation for growth and development [3]. During this time, infants often have a period of receiving a combination of oral and tube feedings. Depending on infants’ GA at birth and medical complications, they may receive tube feedings for at least a week or for an extended period of time (2 weeks or longer). Nurses are primarily responsible for feeding preterm infants. Nurses observe infant behaviors surrounding feeding to assess oral feeding readiness. Yet it is not well understood how these behaviors change over time while infants are receiving tube feedings only and through the transition to oral feedings.

Behaviors surrounding feeding, i.e., behavioral states [3–7], social interactive behaviors [7,8], orally directed behaviors [3,4,6,8] and hunger/satiation behaviors [9,10] are indicators of oral feeding readiness, efficiency and safety [2,4,8,11,12]. White-Traut and colleagues previously described the range and changes in behavioral states and orally directed behaviors immediately prior to oral feeding in clinically stable preterm infants [6]. Awake behavioral states are important because they are correlated with oral feeding success and feeding efficiency in both preterm and full-term infants [2,4]. In full-term infants, increased time spent in awake behavioral states and frequency of orally directed behaviors are related to higher nutritive sucking [4,8,13] and non-nutritive sucking organization [14,15]. Evidence further suggests that increased frequency of orally directed behaviors is associated with increased time spent in awake behavioral states and may mediate improved nutritive sucking organization [5].

Additionally, altered behaviors surrounding feeding may result in slow oral feeding progression and less optimal parent-child interaction during initial Neonatal Intensive Care Unit (NICU) hospitalization [2,12]. When infants experience oral feeding difficulty, their capacity for social interaction during feeding may be compromised, leading to less optimal parent-infant interaction [16,17]. During infancy, oral feeding holds the central opportunity for infant growth and development of enduring positive mother-infant interaction [2,8]. Most healthy full-term and preterm infants experience the pleasurable sensations of smell, taste, touch, and social interaction associated with oral feeding [18,19]. They establish the recurring cycle of hunger, oral feeding, and satiation in association with transition of behavioral states from sleep to awake, and exhibition of orally directed behaviors [3,4]. On the other hand, critically-ill-preterm infants often experience traumatic stimuli that circumvent the oral cavity such as insertion of oral gastric tubes, intubation, plastic tastes and smells, and frequent suctioning [3,20]. All of these may drastically alter their behaviors surrounding feeding. Additionally, these infants may require tube feedings for an extended time, contributing to non-establishment of hunger-oral feeding-satiation cycles [3]. Given the significant roles of behaviors surrounding feeding in the infant’s ability to oral feed safely and efficiently, it is critical to understand how infants with extended tube feeding exhibit them early on during the initial post-birth NICU hospitalization.

The purpose of this pilot study was to describe the change in behaviors surrounding feeding and with respect to PMA for preterm infants who received extended tube feedings during the NICU hospitalization.

## Materials and Methods

### Study design & participants

We employed a prospective design. The pilot study was approved by the Institutional Review Board and conducted in a Midwestern level IV Neonatal Intensive Care Unit. Eligibility criteria included: Born between 23 to 32 weeks GA, clinically stable, and expected to have at least two weeks of tube feedings during the initial hospitalization. Exclusion criteria included: Diagnosis of necrotizing enterocolitis, sepsis, intraventricular hemorrhage (grade III or IV), periventricular leukomalacia, cardiovascular defects, congenital anomalies of the oral cavity, gastrointestinal defects, or chromosomal abnormalities. We enrolled 35 infants who met the eligibility criteria. One infant was excluded due to the development of exclusion criteria.

### Measures

#### Infant characteristics/risk factors:

Infant sex, race/ethnicity, GA at birth, birth weight, length and head circumference, delivery type, Apgar scores and intrauterine growth restriction diagnosis were obtained from the electronic medical record.

#### Feeding progression and health status during hospitalization and at discharge:

Feeding progression was measured by the duration of tube feedings and duration of oral feeding transition (number of days to progress from first to full oral feeding). Feeding type at the time of evaluation was recorded. Health status during hospitalization was evaluated via bronchopulmonary dysplasia severity, duration of oxygen therapy, and oxygen flow. Health status at discharge was evaluated via weight, duration of hospitalization, oxygen therapy, feeding type, Post-Menstrual Age (PMA) and Chronological Age (CA).

#### Behaviors surrounding feeding:

Behaviors surrounding feeding were evaluated once weekly from 29 to 42 weeks PMA. The videos were segmented and coded by two research assistants (who were blinded to the purpose of the study) using the Mangold Interact 15.1 software (Mangold International, Arnstorf, Germany). The primary research assistant coded 100% of videos. The secondary research assistant re-coded a random 10% to establish inter-rater reliability [21].

#### Behavioral states:

Behavioral states were evaluated using the modified Thoman’s State Scoring System [3,4]. This scoring system includes eight categories of behavioral states: quiet sleep, active sleep, sleep-wake transition, drowsy, quiet alert, active alert, non-alert-waking activity, and fuss/crying. The predominant behavioral state observed for each 15-second interval was recorded.

#### Social interactive behaviors:

Social interactive behaviors were evaluated using Barnard’s indicators [7,8,10]. Social interactive behaviors included two categories: engagement and disengagement behaviors. Engagement behaviors included eye widening, facial brightening, hands open, fingers lightly flexed, searching movements of eyes, facial gaze, mutual facial gaze, smooth cyclic movements of extremities, eyes brightening, and relaxed posture. Disengagement behaviors included hiccup, facial grimace, eyes clenched, gaze aversion, tongue protrusion, finger splay, struggling movements, yawning, or finger extension, hunger posture, crying, whining, fussing, maximal lateral gaze aversion, spitting/vomiting, and halt hand. The frequency of each social interactive behavior observed was recorded for each 15-second interval.

#### Orally directed behaviors:

Orally directed behaviors were evaluated using the Cagan Video Coding System [22]. Any occurrence of mouthing, rooting, tonguing, yawning, sucking-on-tongue, empty-sucking, swipe-at-mouth, hand-to-mouth, and suck-on-hand was noted and recorded. The frequency of each orally directed behavior observed was recorded for each 5-second interval.

#### Hunger/satiation behaviors:

Hunger/satiation behaviors were evaluated using Barnard’s indicators [10]. Hunger behaviors included vocalization, stirring, interest in the pacifier, arms in flexion, large motor movement, head movement, agitated/fussy, tensed posture, crying, eyes open, eyes searching alert or drowsy, head turning to caregiver, and exhibiting orally directed behaviors. Satiation behaviors included arms and legs extended, decreased muscle tone, relaxed posture, relaxed fingers, lack of facial expression, drowsy, and asleep. The frequency of each hunger/satiation behavior observed was recorded for each 15-second interval.

#### Procedure:

Mothers provided written informed consent for their infants’ participation in the study. Infant data were obtained from the medical record. Infants were video recorded once weekly during pre-feeding (30 minutes) and intra-feeding (the length of feeding). The number of video recordings ranged from 2 to 8. Recorded feeding sessions were inclusive of tube feeding only, bottle/breast-feeding only, or both during the transition from tube to full oral feeding.

## Statistical Analysis

We performed data analyses via Stata 13.1 [23] and R 3.6.1 [24] with package lme4 [25]. The eight behavioral state categories were reduced into four categories: sleep, awake, transition and cry [3–5]. We grouped PMA into four categories: 29–31 (*n* = 5), 32–34 (*n* = 25), 35–37 (*n* = 27), and 38–42 (*n* = 8). The relative proportions of each behavioral state category, as well as the frequency of social interactive behaviors, orally directed behaviors, and hunger/satiation behaviors were calculated. For behavioral states, we estimated the difference in the relative proportion of each category (simply denoted as r) comparing two groups with 1 week difference in gestational age or comparing two gestational age groups. For other behaviors, we estimated the log relative risk of the occurrence of each specific behavior (simply denoted as r) comparing two groups with a 1 week difference in PMA or comparing two PMA groups. The effects were estimated using generalized linear mixed models. The trend of each behavior over time or comparison between PMA groups was analyzed separately for pre- and intra-feeding.

## Results

### Infant Characteristics/Risk Factors, Feeding Progression, and Health Status during Hospitalization and at Discharge:

Infants were born at a mean of 28 weeks gestation with 79% delivered by Cesarean section. Mean duration of tube feeding was 58 days and the mean length of transition to oral feeding was 22 days. Fifty-two percent of the infants were diagnosed with moderate bronchopulmonary dysplasia and 82% of the infants were feeding orally at discharge. Results are summarized in table 1.

### Behaviors surrounding feeding during hospitalization

#### Behavioral states:

During the pre-feeding segment, there was an increase over time in the relative proportion of awake (r = .016, p= .007) and cry (r = .007, p= .048) and a significant increased trend with advancing PMA. During the intra-feeding segment, we found a significant change with advancing PMA in the relative proportion of transition (increased trend) and cry (decreased trend). See table 2.

#### Social interactive behaviors:

During the pre-feeding segment, we found an increased frequency over time in facial gaze (r = .36, p= .05), smooth movements (r = .51, p = .03), eye clenched (r = .12, p= .03), gaze aversion (r = 1.01, p= .00), tongue protrusion (r = .15, p= .02), and fussing (r = .22, p= .00). During the intra-feeding segment, we found an increased frequency over time in whining (r = 1.62, p= .04) and fussing (r = .23, p= .00), and decreased frequency in eye widening (r = −.32, p = .01), eye searching (r = −.13, p= .00), and yawning (r = −.28, p= .00). During the pre-feeding segment, there was an increased trend in fussing with advancing PMA. During the intra-feeding segment, there was also in an increased trend in fussing and a decreased trend in yawning with advancing PMA. See table 3.

#### Orally directed behaviors:

During the pre-feeding segment, we found an increased frequency over time in mouthing (r = .060, p= .00) and significant fluctuation in the frequency of hand-to-mouth with advancing PMA. See table 4.

#### Hunger/Satiation behaviors:

During the pre-feeding segment, we found an increased frequency over time in vocalization (r = .33, p= .00), stirring (r = .26, p= .01) and interest in the pacifier (r = .38, p= .00). During the intra-feeding segment, we found an increased frequency over time in vocalization (r = .29, p= .00). During the pre-feeding segment, there was a significant increased trend in vocalization and interest in the pacifier with advancing PMA. During the intra-feeding segment, there was in increased trend in vocalization with advancing PMA. See table 5.

#### Inter-rater reliability:

Inter-rater reliability is moderate to high when the Kappa value is .4 or higher. The Kappa values for the behaviors surrounding feeding data ranged from 0.41 – 0.67, demonstrating at least moderate inter-rater reliability.

## Discussion

To our knowledge, this was a first pilot study to comprehensively describe the changes in behaviors surrounding feeding over time and with respect to advancing PMA for preterm infants who received extended tube feedings during the NICU hospitalization. Changes with advancing PMA were identified for a limited number of behaviors surrounding feeding. Overall, there was an increasing trend over time for awake behavioral states, multiple social interactive behaviors, and mouthing during the pre-feeding segment. Infants demonstrated an increasing trend over time in vocalization during both pre- and intra-feeding segments.

There was a significant change in awake and crying behavioral states with advancing PMA, peaking between 38–42 weeks PMA. Behaviors surrounding feeding are related to feeding readiness and efficiency [2,4,8,12]. For example, infants who demonstrate awake behavioral states also have improved feeding efficiency and sucking organization [4,5,26]. Of note, the research by Park et al. showed that infants who had delays in the development of oral feeding progression exhibited a slower rate of active and quiet sleep during the daytime hours when compared to infants with typical feeding progression [27]. Infants with delayed oral feeding progression also exhibited increased awake behavioral states during nighttime hours. Further research is warranted to understand how day/night cycles or an intervention to improve awake behavioral states prior to feeding might help infants to better regulate their behaviors surrounding feeding.

There remains limited understanding regarding the maturation of social interactive behaviors surrounding feeding in preterm infants. In our sample, both engagement and disengagement behaviors increased over time during pre-feeding segment, including facial gaze, smooth movements, eye clinch, gaze aversion, tongue protrusion, and fussing. During the intra-feeding segment, we found that eye widening, eye searching, and yawning decreased while whining and fussing increased over time. These behaviors have been associated with feeding preparedness in older infants [28,29]. In addition, these behaviors may be suggestive of infants’ learning to regulate their behaviors. Preterm infants may only be able to sustain eye contact for short periods of time, thus they need to close their eyes during feeding as a mean to regulate their behavior. We speculate that the decrease in eye widening and eye searching is suggestive that preterm infants may attempt to regulate their behaviors to reduce environmental stimuli and support feeding. The facial grimacing, eyes clenched, tongue protrusion, and fussing might be indicators of stress; or infants’ learning to regulate their behaviors during social interaction to reduce their stress. Overall, the findings are likely indicative of preterm infants developing capacity for regulating behaviors [7] surrounding feeding over time and with advancing PMA.

Orally directed behaviors are important antecedents of oral feeding success [2–5]. Demonstration of orally directed behaviors prior to feeding predicts feeding efficiency [2,11,30]. Kirk and colleagues [30] postulated that natural display of orally directed behaviors was an important prerequisite for oral feeding. Prior research with full-term infants indicated that orally directed behavioral cues are critical indicators of organized oromotor neurobehaviors, and thus may play a significant role in the infant’s ability to communicate readiness to feed as well as sustain successful breast feeding [2,8]. Additionally, as the infants matured, they demonstrated a fluctuation in the frequency of hand-to-mouth with advancing PMA. Infants in our study demonstrated an increase over time in the frequency of mouthing during the pre-feeding segment when they were between 31 and 37 weeks, however, a decrease trend was observed after 37 weeks. There were six infants remaining in the NICU between 38 to 42 weeks. They were still receiving oxygen. Thus, these infants may have been more medically fragile during this time period, which was demonstrated by the decrease in mouthing at these later ages. Key clinical indicators of oral feeding readiness include rooting, tonguing, yawning, sucking-on-tongue, empty-sucking, swipe-at-mouth, or suck-on-hand. However, we did not identify any change over time or with advancing PMA during the pre-feeding segment for any of these orally directed behaviors. Our finding suggests that mouthing and hand-to-mouth may provide a more precise and clinically relevant assessment.

Infants in our study also demonstrated an increased frequency over time in vocalization, stirring, and interest in the pacifier during pre-feeding segment. With advancing PMA, we observed a significant increased trend in vocalization pre- and intra-feeding. Our findings are suggestive that infants are capable of exhibiting changes in the frequency of limited orally directed behaviors and hunger/satiation as they are preparing to feed and with advancing PMA. However, it was unexpected that several other important orally directed behaviors and hunger/satiation behaviors did not change surrounding feeding. The lack of change in some orally directed and hunger/satiation behaviors may jeopardize the infant’s ability to safely and efficiently oral feed. Future research of interventions that target the increased frequency of these behaviors to support safe and efficient oral feeding is warranted.

There are several limitations in this study. While all infants were video recorded for a minimum of two weeks during tube feeding and prior to starting oral feeding, the total number of weeks each infant participated varied depending on GA at birth, age at first video recording session, health status, and age at discharge. Therefore, the number of weeks each infant provided data was not consistent across this sample (range from 1 to 8 with a median of 4 visits per child).The statistical estimates were unstable before 30 weeks and after 40 weeks PMA due to the limited sample size for these ages. Overall, the pilot study included a small sample size (N = 34) and the descriptive design limits generalizability of findings.

## Conclusion

Infants as young as 28 weeks PMA demonstrated feeding behaviors pre- and intra-feeding while receiving extended tube feeding and during the transition from tube to full oral feeding. Feeding behaviors vary among infants and change significantly over time with more mature PMA. Although the feeding behaviors may be subtle, it is crucial for clinicians and researchers to carefully consider infant feeding behaviors during assessment when starting and ending a feeding session, ensuring safe and efficient feeding. Understanding infants’ capacity during the transition from tube feeding to oral feeding may lead to changes in practice as clinicians will understand how these additional indicators support a more comprehensive assessment of readiness for oral feeding and the relationship to efficient oral feeding. Infants who receive tube feedings for an extended time period while hospitalized are at risk for oral feeding difficulty and poor growth and development. Identification of the altered feeding behaviors and when or how oral feeding difficulty emerges will serve to support the implementation of a positive feeding experience during the tube feeding only period and over the transition from tube to full oral feeding, improving clinical practice and reducing cost.

Although this was a pilot study, the results lead to an important question that warrants future research. Future research should include the measurement and evaluation of sucking metrics and stress-related biobehavioral markers in relationship to the four types of behaviors surrounding feeding presented in this report. This line of inquiry would advance understanding of how the behavioral indicators surrounding feeding and over time may impact sucking metrics, and thus overall oral feeding skills and efficiency. Research with a larger sample would allow for the analysis of covariates such as GA at birth, birth weight, infant sex, race/ethnicity, delivery type, Apgar scores, intrauterine growth restriction, health status during hospital stay, oxygen requirements, and age at first oral feeding. Finally, prospective randomized-controlled-trials comparing developmental interventions in relationship to behaviors surrounding feeding, sucking metrics, and stress-related biobehavioral markers might further serve to identify which interventions impact each of the four types of feeding behavioral measures and provide additional benefits such as improving oral feeding efficiency.

## Figures and Tables

**Table 1:** Infant Characteristics (N = 34).

Variables	%	Mean (SD)
Infant sex		
Male	47.06	
Female	52.94	
Race/Ethnicity		
Black	35.29	
Latinx	2.94	
White	58.82	
Other	2.94	
Gestational age at birth (weeks)		28.15 (2.30)
Birthweight (gram)		1066.18 (308.14)
Length at birth (cm)		35.82 (3.74)
Head circumference at birth (cm)		25.71 (2.99)
Type of delivery		
Spontaneous vaginal	17.65	
Non-spontaneous vaginal	2.94	
Cesarean section	79.41	
1-minute Apgar score		4.38 (2.47)
5-minute Apgar score		6.85 (1.64)
Intra uterine growth restriction		
No	91.18	
Yes	8.82	
Duration of tube feeding (days)		58.16 (22.05)
Duration of oral feeding transition (days)		22.34 (9.84)
Bronchopulmonary Dysplasia severity		
None	23.53	
Mild	2.59	
Moderate	52.94	
Severe	2.94	
Duration of oxygen therapy (days)		44.5 (34.65)
Oxygen flow at observations (133 observations)		
Room air	34.59	
More than 1 L	65.41	
Feeding type at observations		
Oral	12.69	
Tube	47.01	
Combination	4.30	
Weight at discharge (gram)		2629.56 (459.17)
Duration of hospitalization (days)		69.06 (24.54)
Oxygen therapy at discharge		
No	52.94	
Yes	47.06	
Feeding type at discharge		
Oral	82.35	
Tube	0	
Combination	17.65	
Post-menstrual age at discharge (weeks)		38.18 (1.92)
Chronological age at discharge (weeks)		9.73 (3.42)

**Table 2:** Behavioral states: Generalized linear mixed model analysis.

Pre-feeding	*r*_32–34_	*r*_35–37_	*r*_38–42_	*p*
Awake	.04	.02	.22	**.04**
Cry	.02	.02	.06	**.00**
Intra-feeding	r32–34	r35–37	r38–42	*p*
Transition	−.02	−.03	.06	**.04**
Cry	.00	.02	.00	**.00**

*Note*. Baseline is 29–31 weeks PMA. r = relative proportion for each respective PMA group.

**Table 3:** Social interactive behaviors: Generalized linear mixed model analysis.

Pre-feeding	*r*_32–34_	*r*_35–37_	*r*_38–42_	*p*
Fussing	1.28	1.94	2.12	**.00**
Intra-feeding	r32–34	r35–37	r38–42	*p*
Fussing	2.87	3.54	3.52	**.00**
Yawning	−.54	−1.02	−2.59	**.05**

*Note*. Baseline = 29–31 weeks PMA. r = log relative risk for each respective PMA group.

**Table 4:** Orally directed behaviors: Generalized linear mixed model analysis.

Pre-feeding	*r*_32–34_	*r*_35–37_	*r*_38–42_	*p*
Hand-to-mouth	.27	.87	−1.77	.01

*Note*. Baseline = 29–31 weeks PMA. r = coefficient of log relative risk for each respective PMA group.

**Table 5:** Hunger/Satiation behaviors: Generalized linear mixed model analysis.

Pre-feeding	r_32–34_	r_35–37_	r_38–42_	*p*
Vocalization	2.06	2.98	3.32	**.00**
Pacifier Interest	−.21	1.49	2.41	**.01**
Intra-feeding	r32–34	r35–37	r38–42	***p***
Vocalization	3.38	4.25	4.63	**.00**

*Note*. Baseline = 29–31 weeks PMA. r = coefficient of log relative risk for each respective PMA group.
